# Managing Primary Immunodeficiency Immunoglobulin Replacement Therapy‐Related Adverse Events With Recombinant Human C1 Esterase Inhibitor Prophylaxis: A Case Report

**DOI:** 10.1002/ccr3.71988

**Published:** 2026-02-05

**Authors:** Douglas H. Jones, Heidi Memmott

**Affiliations:** ^1^ Rocky Mountain Allergy, Asthma, and Immunology Salt Lake City Utah USA; ^2^ Pharming Healthcare, Inc Warren New Jersey USA

**Keywords:** chronic diseases, emergency medicine, general medicine, immunology

## Abstract

Immunoglobulin replacement therapy (IRT) for primary immunodeficiency reduces infection risk and subsequent complications and can be lifesaving. However, IRT can cause severe systemic adverse events (AEs) that may limit adequate dosing. These AEs may be caused, in part, by activation and/or consumption of complement proteins, thereby lowering C1 esterase inhibitor (C1‐INH) levels. Data suggest that C1‐INH administration prior to intravenous immunoglobulin (IVIG) may reduce IVIG‐related AEs. This case describes an adult with common variable immunodeficiency unable to tolerate IRT therapy (subcutaneous immunoglobulin [SCIG] 20% solution once weekly). She experienced AEs of severe neuropathy, described as burning and pins‐and‐needles sensation in the extremities and muscle twitching for several days post‐treatment. Dose decreases of SCIG to 0.5 g did not improve the AE profile. Inability to tolerate IRT caused suboptimal dosing and inadequate primary immunodeficiency management, resulting in hospitalizations for pneumonia and sepsis. A trial of recombinant human C1‐INH 4200 U was administered intravenously over approximately 5 min, 1 h prior to SCIG 1 g (Day 1). This dose was well tolerated with minimal AEs reported. SCIG 3 g was administered on Days 2 and 3 with no AEs reported. By continuing routine recombinant human C1‐INH 4200 U prophylaxis, the patient was able to tolerate the recommended dose of SCIG 20 g once weekly without the debilitating neuropathy and other AEs previously experienced with SCIG alone. This case suggests that a patient with IRT‐related AEs may benefit from C1‐INH replacement therapy prior to SCIG/IVIG administration to improve tolerability.

## Key Clinical Message

This case suggests that a patient with severe immunoglobulin replacement therapy‐related adverse events might benefit from C1 esterase inhibitor (C1‐INH) replacement therapy prior to immunoglobulin replacement therapy for primary immunodeficiency. In appropriate patients, a trial of C1‐INH replacement therapy as part of an adverse event management strategy might improve tolerability.

## 1 Introduction

Immunoglobulin replacement therapy (IRT) reduces the risk of infection and subsequent complications and can be a lifesaving, long‐term therapy for patients with primary immunodeficiency (PI), such as common variable immunodeficiency (CVID) [1]. However, IRT administration can cause systemic adverse events (AEs), whether it is administered intravenously (intravenous immunoglobulin [IVIG]) or subcutaneously (subcutaneous immunoglobulin [SCIG]) [1, 2, 3]. It has been hypothesized that administration of IRT may cause activation/consumption of complement proteins, thereby lowering C1 esterase inhibitor (C1‐INH) levels [2, 4]. C1 esterase inhibitor is a key regulator of the complement and contact systems and also plays a role in inhibition of serine proteases of the coagulation and fibrolytic pathways [5]. A study including healthy volunteers showed that a major driver of C1‐INH metabolism is likely the complement and contact system [6]. In addition, preliminary data suggest that at least some patients with CVID have low levels of C1‐INH [2].

IRT may drive complement activation, leading to low C1‐INH levels and causing systemic AEs [2, 4]. An open‐label, pilot study in adults with CVID (*n* = 16) or polyneuropathy (*n* = 3) suggested that intravenous recombinant C1‐INH administered immediately prior to IVIG infusion can reduce the intensity of IVIG‐related AEs of headache and fatigue [4]. Data are lacking on the impact of subcutaneous administration of IRT on C1‐INH consumption and the potential benefit of C1‐INH supplementation for reducing the intensity or occurrence of IRT‐associated AEs. This report discusses the case of the administration of recombinant human C1‐INH (rhC1‐INH) in an individual with PI who was unable to tolerate SCIG therapy due to severe AEs.

## 2 Case Report

### 2.1 Case History

**FIGURE 1:**
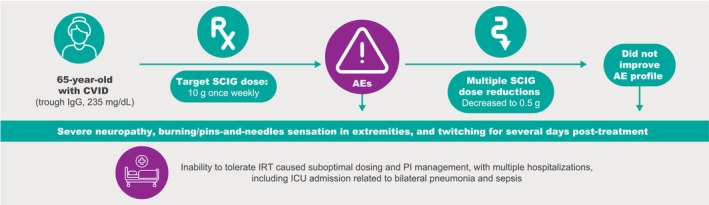
Patient medical history. AE, adverse event; CVID, common variable immunodeficiency; ICU, intensive care unit; IgG, immunoglobulin G; IRT, immunoglobulin replacement therapy; PI, primary immunodeficiency; SCIG, subcutaneous immunoglobulin.

The patient was a 65‐year‐old woman newly diagnosed with CVID (trough immunoglobulin G [IgG], 235 mg/dL) who began treatment with a 35‐g weight‐based dose of immune globulin infusion (human), 10% (Gammagard Liquid, Takeda Pharmaceuticals USA Inc., Lexington, MA). Within hours of receiving half the intravenous dose, the patient presented in the emergency department with nerve pain, severe itching, a rash, tachycardia, and hypertension, and was diagnosed with red man syndrome. Subsequently, she was switched to SCIG (Cuvitru; Takeda Pharmaceuticals USA Inc., Lexington, MA) 10 g once weekly. Within 2 h post‐treatment, the patient experienced severe neuropathy, described as burning/pins‐and‐needles sensation in the extremities, and muscle twitching, all of which lasted for several days post‐treatment. The patient did not report experiencing AEs of edema, headache, or fatigue. Given the ongoing negative impact on the patient's health due to the low IgG levels, her health care provider petitioned the insurance company for approval of a trial of off‐label C1‐INH replacement therapy to determine if the severe adverse effects of IRT could be mitigated. The letter of medical necessity was not approved. Subsequently, the patient had an admission to the intensive care unit for bilateral pneumonia and sepsis (Figure 1).

### 2.2 Treatment

**FIGURE 2:**
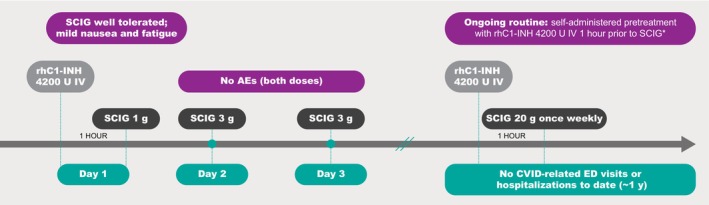
Prophylaxis with C1‐INH replacement therapy may manage SCIG‐related adverse events. *Current regimen during ~1 year period. AE, adverse event; ED, emergency department; IV, intravenous; rhC1‐INH, recombinant human C1 esterase inhibitor; SCIG, subcutaneous immunoglobulin.

Because the suboptimal dosing and discontinuation of SCIG for CVID led to ongoing health issues, a second request for coverage of a trial of C1‐INH replacement therapy was approved by the insurance provider. Single‐dose rhC1‐INH (Ruconest, Pharming Healthcare Inc., Warren, NJ) 4200 U was administered as an intravenous injection, over approximately 5 min, at 1 h prior to SCIG 1 g on Day 1 (Figure 2). The SCIG dose was well tolerated, with only mild nausea and mild fatigue reported and with no symptoms of neuropathy. Subsequently, SCIG 3 g was administered on Days 2 and 3, with no AEs reported. With at‐home, routine rhC1‐INH 4200 U pretreatment (prophylaxis) 1 h prior to SCIG administration, the patient currently is tolerating the recommended dose of SCIG 20 g once weekly without the debilitating neuropathy and other AEs previously experienced with SCIG alone.

## 3 Discussion

This is the first known report of C1‐INH replacement therapy for the treatment of severe AEs related to SCIG administration in a patient with PI. Pretreatment (prophylaxis) with rhC1‐INH allowed administration of the recommended IRT dose in a patient with PI who otherwise could not tolerate this standard‐of‐care, lifesaving therapy. The potential benefit of incorporating prophylaxis with rhC1‐INH into the PI management strategy for CVID in this patient was shown by an improved SCIG tolerability profile and reduction in CVID‐related emergency department visits and hospitalizations during the approximately 1 year of the rhC1‐INH prophylactic regimen. Intravenous rhC1‐INH is indicated for acute treatment of hereditary angioedema attacks in adolescents and adults [7]. Hereditary angioedema is a genetic condition that is most often caused by a deficiency or dysfunction of C1‐INH, which affects multiple enzymes involved in the complement (C1r, C1s, mannose‐binding lectin‐associated serine protease 1 and 2), contact‐system (plasma kallikrein, coagulation factor XIIa), and fibrinolytic (plasmin) pathways [8, 9]. Administration of rhC1‐INH provides a direct replacement for low levels of C1‐INH. In the treatment of hereditary angioedema attacks, the recommended dosing for rhC1‐INH is body‐weight based (< 84 kg [50 U/kg]; ≥ 84 kg [4200 U]) [7]. In the case reported here, the same weight‐based dosing regimen was used, with the patient receiving rhC1‐INH 4200 U. The duration of effect of rhC1‐INH as a pretreatment (prophylaxis) for immunoglobulin‐related AEs was 3 or more days, which is consistent with the profile observed in patients with hereditary angioedema [10].

A study by Melamed et al. [2] found that 37.5% of 80 patients newly diagnosed with CVID had low levels of C1‐INH protein (mean, 16.7 mg/dL; normal range, 21–39 mg/dL) and/or reduced C1‐INH enzyme function (mean [SD], 37.3% [12%]; lower limit of normal, 67%). Levels of complement C4 were found to be within normal range [2]. That study reported that patients with lower serum C1‐INH levels often experienced edema and had AEs of headache and fatigue after IVIG treatment [2]. In the current case report, the patient experienced severe, distinct symptoms after SCIG treatment to those previously reported by Melamed et al. [2, 4] The cause of immunoglobulin‐related AEs is not fully understood, but it may be related to complement activation (e.g., interaction between antibodies administered and circulating antigens [e.g., infectious agents] in the patient) or activating molecules in the immunoglobulin formulation [1, 4, 11].

Strengths of the approach to this case include the proactive and systematic process used to manage IRT‐related AEs in an individual with PI, including the persistence of health care staff to obtain insurance coverage of this investigational pretreatment (prophylaxis). Limitations of the approach include incomplete details around the full negative impact of suboptimal dosing of SCIG that occurred prior to implementation of rhC1‐INH prophylaxis and lack of quantitative data on IgG (trough) and C1‐INH levels, functional C1‐INH, and emergency department visits/hospitalizations for the patient during the time frame discussed. Overall, this case suggests that a patient with severe IRT‐related AEs may benefit from C1‐INH replacement therapy, such as rhC1‐INH, prior to SCIG or IVIG treatment for PI. Although this case is considered hypothesis generating and future research is needed, health care providers should be aware of this potential AE management strategy.

## Author Contributions

**Douglas H. Jones:** conceptualization, data curation, methodology, writing – original draft, writing – review and editing. **Heidi Memmott:** writing – review and editing.

## Funding

This work was supported by Pharming Healthcare Inc., Funding for editorial/medical writing assistance.

## Consent

Written informed consent was obtained from the participant for publication of this case report.

## Conflicts of Interest

D.H.J. reports being a speaker and/or consultant for AstraZeneca, Pharming Healthcare Inc., Regeneron Pharmaceuticals Inc./Sanofi, and Takeda Pharmaceutical Co. Ltd. H.M. is an employee of Pharming Healthcare Inc.

## Data Availability

All available data are included in the article. Further inquiries can be directed to the corresponding author.
